# Study of the predictive value of testosterone in androgen deprivation therapy for metastatic hormone-sensitive prostate cancer—the dual clinical research center for western and eastern China

**DOI:** 10.3389/fendo.2025.1630862

**Published:** 2025-10-17

**Authors:** Dongsheng Ma, Xiaoguang Zhang, Jianhong Xi

**Affiliations:** ^1^ Department of Reproductive Medicine, The Affiliated Bozhou Hospital of Anhui Medical University, Bozhou, China; ^2^ Department of Reproductive Medicine, The People’s Hospital Bozhou, Bozhou, China; ^3^ Reproductive Male Laboratory, The People’s Hospital Bozhou, Bozhou, China; ^4^ Department of Urology, The First Affiliated Hospital of Xinjiang Medical University, Urumqi, China

**Keywords:** prostate cancer, testosterone, androgen deprivation therapy, metastatic hormone sensitive prostate cancer, testosterone response

## Abstract

**Objective:**

This study aims to investigate the association between early testosterone (T) response to androgen deprivation therapy (ADT) and clinical outcomes in metastatic hormone-sensitive prostate cancer (mHSPC).

**Methods:**

This retrospective cohort study analyzed 366 mHSPC patients treated at The People’s Hospital Bozhou and The First Affiliated Hospital of Xinjiang Medical University. The participants were stratified by 1-month testosterone response: response group (T < 50 ng/dL) and non-response group (T > 50 ng/dL). The response group was further subdivided into ultra-low (T < 20 ng/dL) and low (20–50 ng/dL) response groups. Comparative analyses of baseline characteristics, progression to metastatic castration-resistant prostate cancer (mCRPC), and survival outcomes were carried out.

**Results:**

No significant intergroup differences were observed in Gleason score, tumor stage, prostate volume, initial PSA, PSA density, perineural invasion, visceral metastasis, or hazard level (all *P* > 0.05). However, the T non-response group exhibited a higher tumor load prevalence (76.77% vs. 60.10%, *P* = 0.004). The T non-response group demonstrated shorter mCRPC progression time (13.38 ± 8.88 vs. 20.40 ± 11.91 months, *P* < 0.001), though no difference emerged between the T ultra-low and low response subgroups (20.59 ± 11.91 vs. 20.86 ± 12.19 months, *P* = 0.876). Survival analysis revealed superior 3-year survival in T responders (*P* = 0.024), with T ultra-low responders showing significant advantages in both overall survival (*P* = 0.010) and 3-year survival (*P* = 0.001) compared to T low responders.

**Conclusion:**

Ultra-low T levels (<20 ng/dL) after 1-month ADT can be used as a reference standard for predicting survival outcomes and may guide treatment optimization in mHSPC.

## 1 Introduction

Prostate cancer (PCa) represents a prevalent epithelial malignancy of the male genitourinary system, characterized by an insidious onset, complex tumor biology, and heterogeneous clinical features. As an immunologically “cold” tumor, it frequently exhibits endocrine resistance, immune evasion, and a propensity for osteogenic bone metastases. According to Global Cancer Statistics 2022, PCa ranks as the fourth most commonly diagnosed cancer worldwide (1,466,700 cases, 7.3% of total) and the eighth leading cause of cancer-related mortality among men (396,800 deaths, 4.1%). Among malignant tumors in men, prostate cancer is at the top of the list in terms of both incidence and prevalence, and there are obvious regional and racial differences (1, 2). During the same period, China’s cancer statistics show that the age-standardized incidence rate (ASIR) of PCa has increased significantly, and the mortality rate is the seventh highest among malignant tumors in men, with a rising trend year by year. The 5-year survival rate in China (66.4%) remains substantially lower than that in Western developed nations (99.5%) (3) despite the incidence and mortality rates remaining below global averages. This disparity is further exacerbated by significantly elevated age-standardized mortality rates and years of life lost (YLL) across both urban and rural Chinese populations (4). While the proportion of cancer burden attributable to modifiable risk factors is projected to decline, smoking, physical inactivity, and inadequate fruit intake continue to represent significant risk factors for cancer mortality (5). The established risk profile for PCa includes non-modifiable factors (age, racial background, family history, and genetic predisposition) alongside potentially modifiable elements (physical activity deficiency, smoking, and obesity) (6, 7). Metastatic PCa is clinically stratified into metastatic hormone-sensitive prostate cancer (mHSPC) and metastatic castration-resistant prostate cancer (mCRPC) based on response to androgen deprivation therapy (ADT). Testosterone (T), the primary male sex hormone, plays crucial roles in metabolic regulation, spermatogenesis, sexual function, and cardiovascular health. Although the aging of the male-specific gonad, testis, is relatively delayed compared to the female-specific gonad, ovarian function, testicular function is severely affected with age, and advanced age adversely affects the hypothalamic–pituitary–gonadal axis (HPGA) and testicular interstitial cells (Leydig cells). Thus, this leads to a physiological decrease in testosterone, which has a negative impact on male reproductive health, especially for prostate hyperplasia and PCa in men of advanced age. Over the past decades, there has been controversy surrounding the relationship between testosterone and PCa. The prostate, as a male urogenital organ, is characterized by a high dependence on and sensitivity to testosterone, which declines physiologically with age, but there seems to be a blurring of the boundaries between the pathological “threshold” or pharmacological “castration” level of PCa. Despite numerous attempts to characterize the relationship between testosterone dynamics and mHSPC progression across the lifespan, high-quality clinical evidence remains insufficient to fully elucidate these mechanisms. Current unmet needs include validated biomarkers to guide therapeutic decisions in mHSPC. Although recent studies suggest testosterone’s potential as a prognostic and predictive marker, its clinical implementation remains controversial and asynchronous with prostate-specific antigen (PSA) monitoring. This study investigates whether differential testosterone suppression thresholds (20 ng/dL vs. 50 ng/dL) during ADT yield distinct survival benefits and progression outcomes in mHSPC, particularly focusing on early testosterone response at 1 month after treatment initiation.

## 2 Methods

### 2.1 Objectives of the study

This retrospective cohort study utilized data from prostate cancer registries at two tertiary medical centers: The People’s Hospital Bozhou (The Affiliated Bozhou Hospital of Anhui Medical University) and the Urology Center of The First Affiliated Hospital of Xinjiang Medical University (Xinjiang Clinical Research Center for Urological Diseases) between 2010 and 2024. From an initial pool of 1,185 patients with pathologically or clinically confirmed prostate cancer, 808 cases were excluded based on predefined inclusion and exclusion criteria. Subsequent exclusions included three duplicate entries, seven lost to follow-up, and one patient declining participation at the study endpoint. The final analytical cohort comprised 366 patients with metastatic hormone-sensitive prostate cancer (mHSPC).

### 2.2 Study criteria

#### 2.2.1 Diagnostic criteria

mHSPC definition: Advanced prostate cancer that has not been treated with endocrine therapy or has responded to endocrine therapy at the time of detection of metastases.

According to the CHAARTED study (8), metastatic hormone-sensitive prostate cancer was classified as high or low tumor load. High tumor load was defined as the presence of ≥4 bone metastases (including ≥1 bone metastasis outside the pelvis or spine) or the presence of visceral metastases, and low tumor load was defined as the absence of the abovementioned factors.

According to the LATITUDE study (9), metastatic hormone-sensitive prostate cancer was classified as high or low hazard level, with high hazard level defined as the presence of two of the following three risk factors: Gleason score ≥8, bone metastases ≥3, and the presence of visceral metastases. Low hazard level was defined as having no more than one of these risk factors.

CRPC definition: Serum T < 50 ng/dL under the condition of castration; accompanied by one of the following conditions: (1) biochemical progression: three consecutive 1-week intervals for PSA elevation, two of which were >50% higher than the lowest value and the absolute value of PSA elevation was >2 ng/mL and (2) imaging progression: bone scanning detected two or more new bone metastases or one or more soft tissue lesions (10).

Testosterone escape: Transient or persistent failure of testosterone to reach castrated levels during ADT.

Testosterone flash: A transient rise in testosterone levels, even to a peak, followed by a gradual return to baseline within 2–4 days of the start of treatment with initial LHRH analogue therapy.

#### 2.2.2 Inclusion criteria

(1) Pathologically definite malignant tumor of the prostate (adenocarcinoma), consistent with distant metastasis M_1_ (including distant lymph node metastasis M_1a_, bone metastasis M_1b_, and visceral metastasis M_1c_), and not having received any previous ADT; (2) ADT, represented by LHRHa analogues, was administered within 1 month after the diagnosis of prostate malignancy; and (3) study follow-up cutoff (April 2025) with a follow-up period of >1 year.

#### 2.2.3 Exclusion criteria

Patients were excluded from the study under the following conditions: (1) presence of severe comorbid cardiopulmonary diseases, major psychological disorders (e.g., anxiety, depression), or concurrent malignancies that significantly reduced life expectancy and could potentially lead to study discontinuation or loss to follow-up, (2) incomplete follow-up data or loss to follow-up, and (3) patient refusal to participate in the study.

### 2.3 Study method

According to the testosterone response or not after 1 month of ADT treatment, the patients were divided into the following two groups: testosterone response group (T < 50 ng/dL), 267 cases; testosterone non-response group (T > 50 ng/dL), 99 cases. The testosterone response group was subdivided into ultra-low testosterone response group (T < 20 ng/dL) at 175 cases and low testosterone response group (T 20–50 ng/dL) at 92 cases.

### 2.4 Study indicators

Basic indicators: age, nationality, hypertension, diabetes, smoking history, drinking history, metabolic syndrome (Mets).

Treatment indicators: continuity of ADT, cumulative duration of ADT treatment (months), ADT treatment specifications.

Testosterone-related indicators: initial testosterone level, testosterone at 1 month of ADT treatment, testosterone response at 1 month of ADT, testosterone escape, testosterone flash.

The testosterone detection method used in this study is chemiluminescent immunoassay (CLIA).

Tumor-related indicators: perineural invasion, initial PSA, PSA density (PSAD), tumor stage, Gleason score, tumor load, tumor hazard level.

Tumor progression indicator: Overall survival (OS): based on the time of death from any cause and from the time of diagnosis of prostate cancer to the time of disease progression or other causes of death, predominantly due to tumor progression or tumor-related complications. It is also divided into 3-year survival time and 5-year survival time according to the study follow-up time. Calculated from the date of PCa diagnosis to the study deadline, the 3-year and 5-year survival time need to meet the 3-year and 5-year follow-up time respectively.

Time to progression to mCRPC: interval from the start of ADT treatment to the diagnosis of mCRPC.

### 2.5 Quality control

1. Data collection: The subjects were screened according to the diagnostic criteria, inclusion criteria, and exclusion criteria, and all data of the study subjects were collected independently by the dual clinical research center through standardized data collection procedures and the electronic data capture (EDC) system.

2. Data quality control: Segment quality control and endpoint quality control were performed on the data management processes involved in this study.

3. Data proofreading: The data were cross-randomly audited by a third party, and the dual-clinical research center were homogeneous in key points such as inclusion criteria, exclusion criteria, and follow-up plan.

### 2.6 Ethical review

The study complied with the principles of the Declaration of Helsinki and was approved by the medical ethics committees of the medical institutions affiliated with the two clinical research centers [ethics approval numbers: BYLS2024-147 (Medical Ethics Committee of The People’s Hospital Bozhou) and K202504-46 (Medical Ethics Committee of The First Affiliated Hospital of Xinjiang Medical University)].

This study is a retrospective study. The study subjects or their authorized relatives have been informed or provided with supplementary explanations regarding the purpose of the study and the potential benefits and risks to patients, and informed consent has been obtained from the study subjects or their authorized relatives. This study utilized clinical information from patients, and personally identifiable information has been coded and kept confidential.

### 2.7 Statistical methods

Statistical analyses were performed using SPSS version 22.0. Continuous variables following normal distribution were expressed as mean ± standard deviation and compared using *t*-test; non-normally distributed variables were reported as median (interquartile range) and analyzed with the Mann–Whitney *U* test. Categorical variables were presented as frequencies (percentages) and compared using the *χ*² test. Survival outcomes were evaluated by using Kaplan–Meier analysis with log-rank testing for group comparisons. To mitigate potential confounding effects, propensity score matching (1:2 ratio) was performed using logistic regression based on clinically relevant covariates. Variables demonstrating a significant association (*P* < 0.05) in univariate logistic regression were incorporated into multivariate logistic regression models to identify independent predictors while adjusting for confounders. A two-sided α level of 0.05 defined statistical significance.

### 2.8 Study flowchart

**Figure 1:**
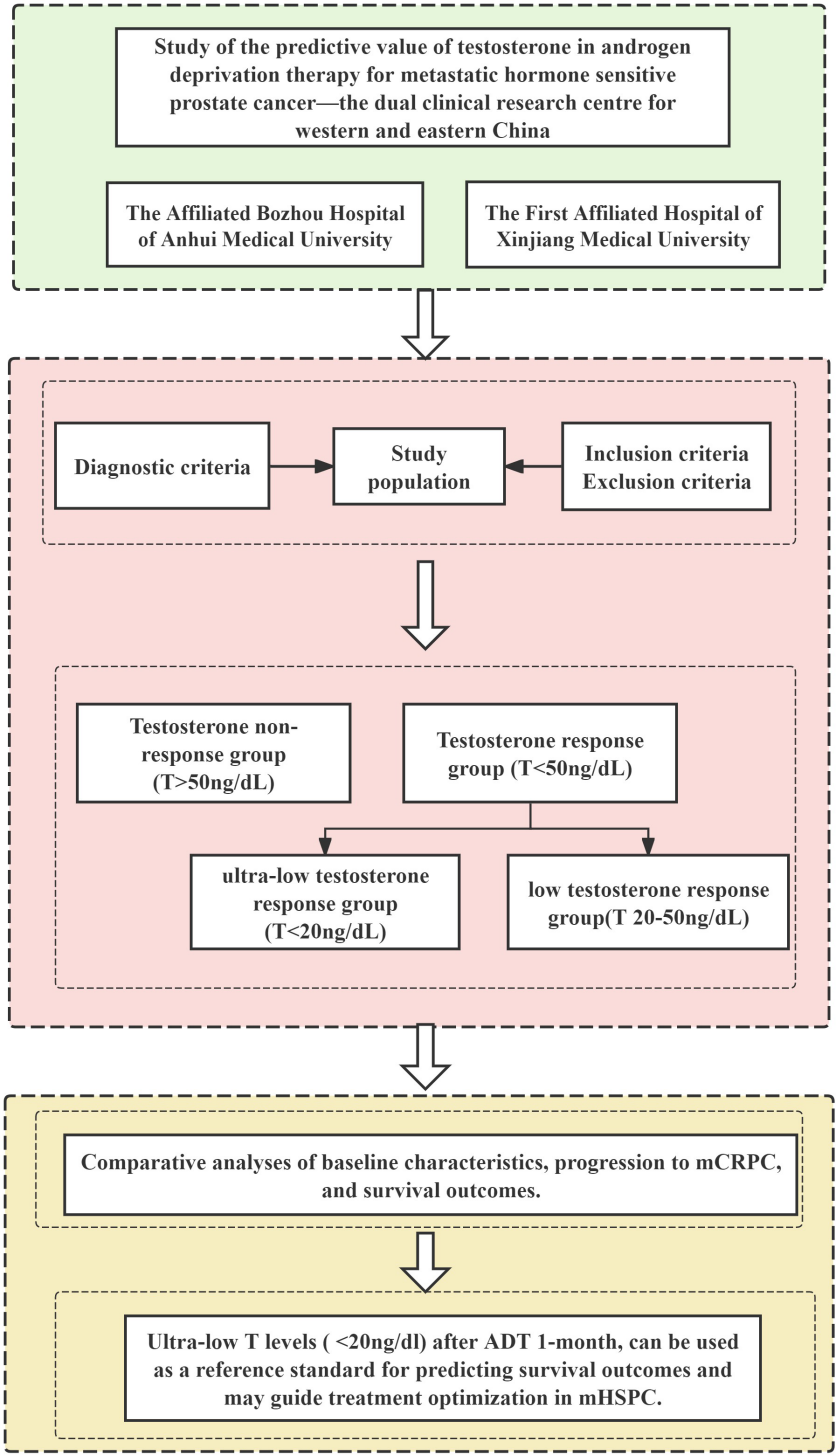
Study flowchart.

**Figure 1** outlines the study design, beginning with patient identification from dual center databases. After applying predefined inclusion/exclusion criteria, eligible mHSPC patients were stratified by 1-month testosterone response post-ADT initiation. Subsequent analyses compared baseline characteristics, progression to mCRPC, and survival outcomes, ultimately validating ultra-low testosterone as a prognostic biomarker for treatment optimization.

## 3 Results

### 3.1 Comparison of demographics and baseline characteristic propensity scores between the two groups

The demographics and baseline characteristics of the study population stratified by group (T non-response group, *N* = 99; T response group, *N* = 267) were propensity score matched (1:2) on patient demographics and baseline characteristics and other relevant baseline variables such as age, smoking history, drinking history, hypertension, diabetes, and metabolic syndrome before and after matching (see **Table 1**).

**Table 1:** Demographics and baseline characteristics between the two groups.

Variables	Level	Before matching				After matching			
		T response group	T non-response group	*P*	SMD	T response group	T non-response group	*P*	SMD
*N*		267	99			198	99		
Age (years)		71.91 ± 8.63	72.60 ± 8.81	0.404	0.078	73.97 ± 8.04	72.60 ± 8.81	0.229	-0.156
Nationality				0.084	0.224			>0.999	<0.001
	Han	189 (70.79)	79 (79.80)			158 (79.80)	79 (79.80)		
	Minority	78 (29.21)	20 (20.20)			40 (20.20)	20 (20.20)		
Smoking history				0.182	0.168			0.771	0.036
	No	189 (70.79)	77 (77.78)			151 (76.26)	77 (77.78)		
	Yes	78 (29.21)	22 (22.22)			47 (23.74)	22 (22.22)		
Drinking history				0.379	0.116			0.883	0.019
	No	237 (88.76)	91 (91.92)			181 (91.41)	91 (91.92)		
	Yes	30 (11.24)	8 (8.08)			17 (8.59)	8 (8.08)		
Hypertension				0.450	-0.089			0.870	-0.020
	No	144 (53.93)	49 (49.49)			100 (50.51)	49 (49.49)		
	Yes	123 (46.07)	50 (50.51)			98 (49.49)	50 (50.51)		
Diabetes				0.009	-0.285			0.195	-0.155
	No	199 (74.53)	60 (60.61)			135 (68.18)	60 (60.61)		
	Yes	68 (25.47)	39 (39.39)			63 (31.82)	39 (39.39)		
Mets				0.058	-0.221			0.622	-0.061
	No	159 (59.55)	48 (48.48)			102 (51.52)	48 (48.48)		
	Yes	108 (40.45)	51 (51.52)			96 (48.48)	51 (51.52)		

SMD, standardized mean difference; Mets, metabolism syndrome.

### 3.2 Comparison of clinical data between the two groups

The cancer clinical data of the study population was stratified by group. No significant differences were observed between groups in Gleason score, T stage, prostate volume, initial TPSA, PSAD, perineural invasion, visceral metastasis, and hazard level. However, T non-response group also had higher proportions of patients with high tumor load than T response group (76.77% vs. 60.10%, *P* = 0.004) (see **Table 2**).

**Table 2:** Cancer clinical data between the two groups.

Characteristics	Group		*χ* ^2^/*Z*	*P*
	T non-response group *N* = 99	T response group *N* = 198		
Gleason score			0.569	0.451
<8	14 (14.14)	22 (11.11)		
≥8	85 (85.86)	176 (88.89)		
T stage			0.258	0.612
<3a	22 (22.22)	39 (19.70)		
≥3a	77 (77.78)	159 (80.30)		
Prostate volume (mL)	62.19 (36.16, 80.00)	55.88 (37.02, 77.03)	-0.862	0.389
Initial TPSA (ng/mL)	156.55 (74.10, 429.22)	194.83 (85.18, 428.90)	-0.468	0.640
PSAD (ng/mL^2^)	2.48 (1.26, 7.47)	3.26 (1.23, 9.49)	-0.864	0.388
Tumor load			8.131	0.004
Low	23 (23.23)	79 (39.90)		
High	76 (76.77)	119 (60.10)		
Perineural invasion			0.009	0.926
No	72 (72.73)	145 (73.23)		
Yes	27 (27.27)	53 (26.77)		
Visceral metastasis			3.264	0.071
No	72 (72.73)	162 (81.81)		
Yes	27 (27.27)	36 (18.18)		
Hazard level			2.387	0.112
Low	29 (29.29)	76 (38.38)		
High	70 (70.71)	122 (61.62)		

The testosterone clinical data of the study population stratified by group. No significant differences were observed between groups in initial testosterone (*P* = 0.566). However, the T response group had longer cumulative ADT duration (14.00 vs. 6.00 months, *P* < 0.001), lower T flash (5.66% vs. 37.37%, *P* < 0.001), lower T escapes for ADT (14% vs. 75%, *P* < 0.001), longer ADT dosing specifications (39.39% vs. 14.14%, *P* < 0.001), and more stable continuous ADT (57.07% vs. 25.25%, *P* < 0.001). The findings highlight significant differences in ADT treatment and testosterone response between the two groups (see **Table 3**).

**Table 3:** Testosterone clinical data between the two groups.

Characteristics	Group		*χ* ^2^/*t*/*Z*	*P*
	T non-response group *N* = 99	T response group *N* = 198		
Initial T (nmol/L)	12.58 ± 5.53	12.98 ± 5.56	-0.575	0.566
Cumulative duration of ADT (months)	6.00 (2.00, 13.00)	14.00 (6.00, 27.25)	-5.497	<0.001
Testosterone flash			49.314	<0.001
No	62 (62.63)	187 (94.44)		
Yes	37 (37.37)	11 (5.56)		
Testosterone escape			148.372	<0.001
No	24 (24.24)	184 (92.93%)		
Yes	75 (75.76)	14 (7.07%)		
ADT dosing specifications			19.685	<0.001
Short (1 month)	85 (85.86)	120 (60.61)		
Long (3 months)	14 (14.14)	78 (39.39)		
Continuity of ADT			26.862	<0.001
No	74 (74.75)	85 (42.93)		
Yes	25 (25.25)	113 (57.07)		

In terms of progression time to mCRPC of the study population stratified by group, the T non-response group has a shorter time than the T response group (13.38 ± 8.88 vs. 20.40 ± 11.91months, *P* < 0.001). The finding highlights significant differences in cancer progression between the two groups (see **Table 4**).

**Table 4:** Time to progression to mCRPC between the two groups.

Characteristic	Overall *N* = 297	Group		*t*	*P*
		T non-response group *N* = 99	T response group *N* = 198		
Progression time to mCRPC	17.78 ± 11.38	13.38 ± 8.88	20.40 ± 11.91	-4.734	<0.001

### 3.3 Comparison of survival between the two groups

The overall study population had a 1-year survival rate of 96.69% (94.59%, 98.84%), a 3-year survival rate of 71.31% (65.65%, 77.45%), and a 5-year survival rate of 58.79% (51.76%, 66.77%). There was no statistical difference in OS between the T non-response and T response groups (*P* = 0.114). This finding revealed that there was no significant difference in overall survival rates (see **Table 5**).

**Table 5:** Kaplan–Meier estimates for survival rates between the two groups.

Characteristics	1 year	3 years	5 years	*P*
Overall	96.69% (94.59%, 98.84%)	71.31% (65.65%, 77.45%)	58.79% (51.76%, 66.77%)	
Group				0.114
T non-response group	94.05% (89.12%, 99.25%)	64.52% (54.14%, 76.89%)	52.70% (41.29%, 67.26%)	
T response group	97.87% (95.83%, 99.96%)	74.27% (67.75%, 81.42%)	61.27% (52.69%, 71.25%)	

**Figure 2:**
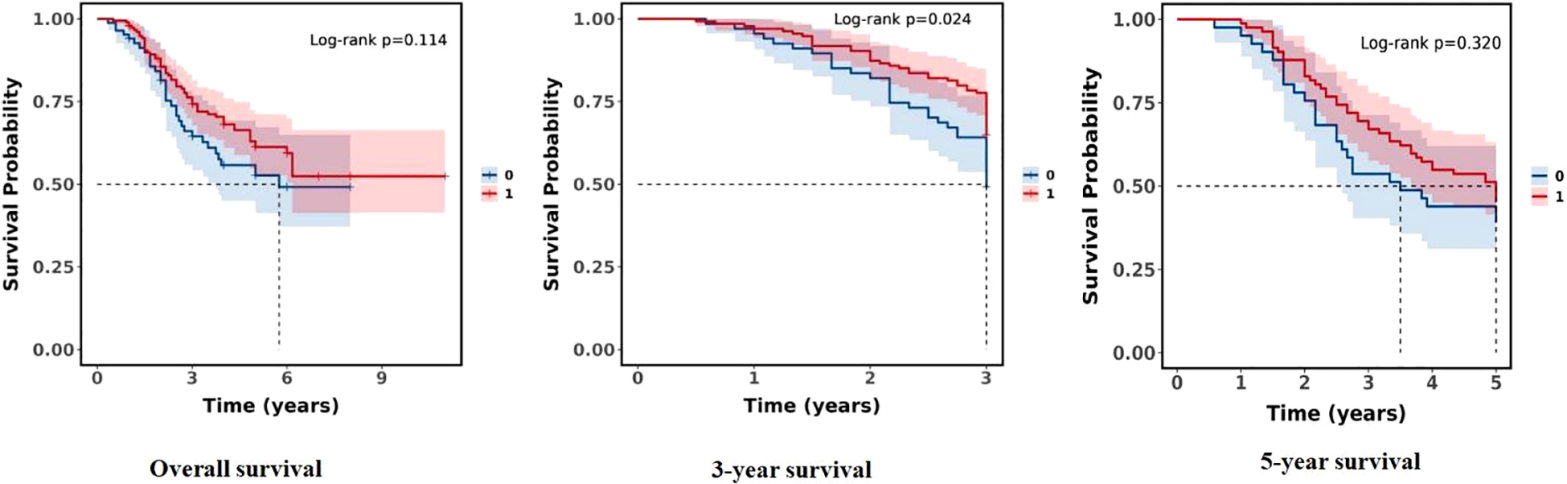
Comparison of survival rates between the two groups (0:T non-response group; 1:T response group).

In terms of survival rates of the study population stratified by two groups (T non-response group and T response group), there was no statistical difference between the two groups in overall survival and 5-year survival (*P* = 0.114, *P* = 0.320, both *P* > 0.05), but there was a statistically significant difference in 3-year survival (*P* = 0.024), and the T response group had a significant survival advantage over the T non-response group in the 3-year survival rate. The findings imply a significant difference in short-term survival between the two groups (see **Figure 2**).

### 3.4 Univariate and multivariate logistic regression analysis of testosterone no-response influencing factors

The multivariate regression analysis revealed significant associations between no variables and the testosterone no-response outcome (see **Table 6**).

**Table 6:** Univariate and multivariate analysis of influencing factors (logistic regression).

Characteristic	Univariable			Multivariable		
	OR	95% CI	*P*	OR	95% CI	*P*
Age	0.99	0.96, 1.02	0.499			
Nationality						
Han	1.63	0.93, 2.85	0.086			
Smoking history						
Yes	1.44	0.84, 2.48	0.184			
Drinking history						
Yes	1.44	0.64, 3.26	0.382			
Hypertension						
Yes	0.84	0.53, 1.33	0.450			
Diabetes						
Yes	0.53	0.32, 0.86	0.010	0.50	0.24, 1.03	0.061
Metabolic syndrome						
Yes	0.64	0.40, 1.02	0.059			
Perineural invasion						
Yes	0.93	0.55, 1.56	0.782			
Visceral metastasis						
Yes	0.54	0.31, 0.93	0.027	0.76	0.33, 1.76	0.518
T stage						
≥3a	1.08	0.62, 1.88	0.796			
Gleason score						
≥8	1.41	0.71, 2.80	0.332			
Tumor load						
Yes	0.47	0.28, 0.79	0.005	0.66	0.31, 1.40	0.279
Hazard level						
Yes	0.67	0.41, 1.10	0.115			
Initial TPSA	1.00	1.00, 1.00	0.990			
PSAD	1.00	1.00, 1.00	0.969			
Initial T	1.01	0.97, 1.05	0.757			

OR, odds ratio; CI, confidence interval.

### 3.5 Comparison between testosterone response subgroups

The testosterone response group was subdivided into ultra-low testosterone response group (T < 20 ng/dL) and low testosterone response group (T 20–50 ng/dL) for the subgroup analysis. The baseline characteristics of 267 patients stratified by group (low T response group: *N* = 92; ultra-low T response group: *N* = 175) were also obtained.

The subgroups were not statistically significant for all demographics and baseline characteristics except testosterone flash (*P* = 0.007). Testosterone flash is precisely the result of the lack of a deep decrease in testosterone. Other categorical variables, including tumor load, ADT continuity, and dosing specifications, demonstrated no significant intergroup differences (all *P* > 0.05). These results also confirm the balanced comparability between subgroups (see **Table 7**).

**Table 7:** Demographics and baseline characteristics between subgroups.

Characteristics	Group			*χ* ^2^/*t*/*Z*	*P*
	Overall *N* = 267	Low T response group *N* = 92	Ultra-low T response group *N* = 175		
Initial TPSA (ng/mL)	211 (86, 477)	199 (90, 431)	211 (84, 494)	0.158	0.875
PSAD (ng/mL^2^)	(1, 10)	4 (1, 10)	3 (1, 10)	-0.471	0.638
Initial T (nmol/L)	12.79 ± 5.62	13.26 ± 5.52	12.54 ± 5.68	0.984	0.326
Cumulative duration of ADT (months)	14 (6, 24)	15 (6, 26)	12 (7, 24)	-0.069	0.945
Age (years)	71.91 ± 8.63	71.73 ± 9.77	72.00 ± 8.00	-0.244	0.807
Nationality				1.205	0.272
Han	189 (70.79)	69 (75.00)	120 (68.57)		
Minority	78 (29.21)	23 (25.00)	55 (31.43)		
Smoking history				0.001	0.972
No	189 (70.79)	65 (70.65)	124 (70.86)		
Yes	78 (29.21)	27 (29.35)	51 (29.14)		
Drinking history				0.297	0.586
No	237 (88.76)	83 (90.22)	154 (88.00)		
Yes	30 (11.24)	9 (9.78)	21 (12.00)		
Hypertension				0.127	0.721
No	144 (53.93)	51 (55.43)	93 (53.14)		
Yes	123 (46.07)	41 (44.57)	82 (46.86)		
Diabetes				3.770	0.052
No	199 (74.53)	62 (67.39)	137 (78.29)		
Yes	68 (25.47)	30 (32.61)	38 (21.71)		
Metabolic syndrome				0.535	0.465
No	159 (59.55)	52 (56.52)	107 (61.14)		
Yes	108 (40.45)	40 (43.48)	68 (38.86)		
Perineural invasion				2.886	0.089
No	198 (74.16)	74 (80.43)	124 (70.86)		
Yes	69 (25.84)	18 (19.57)	51 (29.14)		
Visceral metastasis				0.264	0.607
No	222 (83.15)	75 (81.52)	147 (84.00)		
Yes	45 (16.85)	17 (18.48)	28 (16.00)		
T stage				0.291	0.590
<3a	56 (20.97)	21 (22.83)	35 (20.00)		
≥3a	211 (79.03)	71 (77.17)	140 (80.00)		
Gleason score				0.480	0.489
<8	28 (10.49)	8 (8.70)	20 (11.43)		
≥8	239 (89.51)	84 (91.30)	155 (88.57)		
Hazard level				0.001	0.969
Low	102 (38.20)	35 (38.04)	67 (38.29)		
High	165 (61.80)	57 (61.96)	108 (61.71)		
Tumor load				0.097	0.756
Low	105 (39.33)	35 (38.04)	70 (40.00)		
High	162 (60.67)	57 (61.96)	105 (60.00)		
Continuity of ADT				1.954	0.162
No	115 (43.07)	45 (48.91)	70 (40.00)		
Yes	152 (56.93)	47 (51.09)	105 (60.00)		
ADT dosing specifications				0.013	0.908
Short (1 month)	158 (59.18)	54 (58.70)	104 (59.43)		
Long (3 months)	109 (40.82)	38 (41.30)	71 (40.57)		
Testosterone flash				7.301	0.007
No	252 (94.38)	82 (89.13)	170 (97.14)		
Yes	15 (5.62)	10 (10.87)	5 (2.86)		

In terms of progression time to mCRPC, the study population was also stratified by subgroup (20.59 ± 11.91 vs. 20.86 ± 12.19 months, *P* = 0.876). No significant difference in cancer progression was found between subgroups. The finding highlights, at the castrated level, whether or not testosterone is deeply reduced and does not affect cancer progression (see **Table 8**).

**Table 8:** Time to progression to mCRPC between subgroups.

Characteristic	Overall *N* = 267	Group		*t*	*P*
		Low T response group *N* = 92	Ultra-low T response group *N* = 175		
Progression time to mCRPC	20.76 ± 12.05	20.59 ± 11.91	20.86 ± 12.19	-0.156	0.876

The overall study population had a 1-year survival rate of 97.69% (95.47%, 99.95%), a 3-year survival rate of 76.28% (69.77%, 83.39%), and a 5-year survival rate of 61.64% (52.75%, 72.03%). There was a statistical difference in overall survival between low T response and ultra-low T response subgroups (*P* = 0.010). This finding revealed that there was a significant difference in overall survival rates between subgroups (see **Table 9**).

**Table 9:** Kaplan–Meier estimates for survival rates between subgroups.

Characteristic	1 year	3 years	5 years	*P*
Overall	97.69% (95.47%, 99.95%)	76.28% (69.77%, 83.39%)	61.64% (52.75%, 72.03%)	
Group				0.010
Low T response group	97.62% (94.41%, 100.00%)	67.82% (57.96%, 79.35%)	49.67% (37.61%, 65.60%)	
Ultra-low T response group	97.75% (94.72%, 100.00%)	84.55% (76.85%, 93.02%)	76.26% (66.86%, 86.99%)	

**Figure 3:**
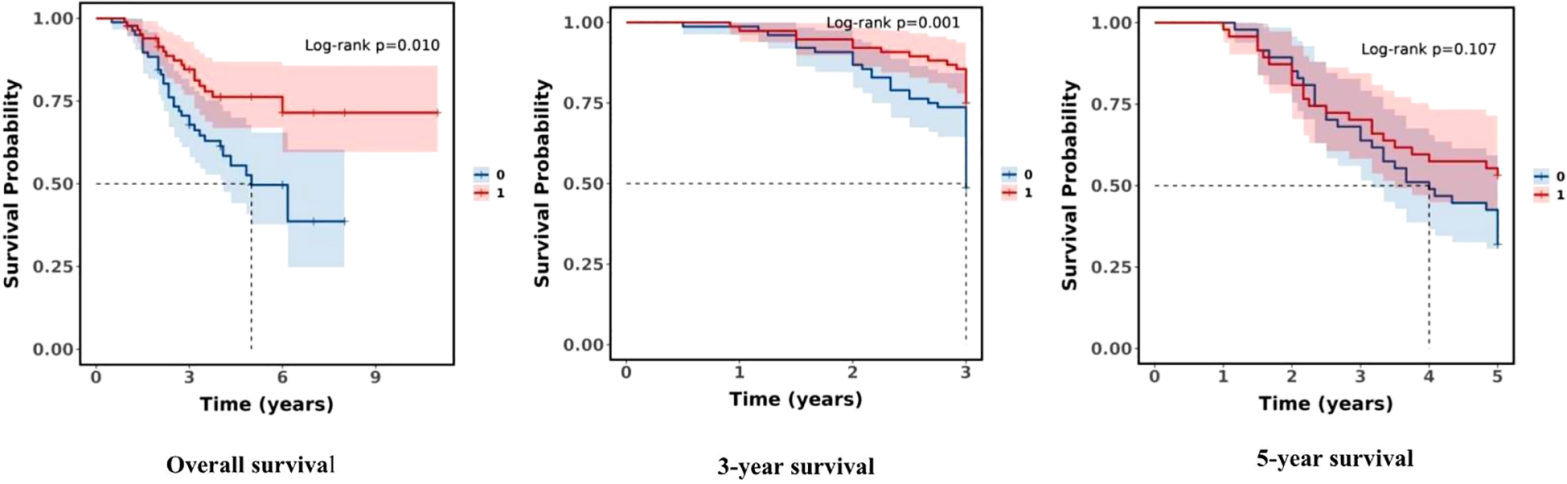
Comparison of survival rates between the subgroups (0:Low T response group; 1:Ultra-low T response group).

The survival rates of the study population stratified by subgroups (ultra-low T response group and low T response group) were also established. There was a statistical difference between the two groups in overall survival and 3-year survival (*P* = 0.010, *P* = 0.001, both *P* < 0.05), but there was no statistically significant difference in 5-year survival (*P* = 0.107), and the ultra-low T response group had a significant survival advantage over the low T response group in overall survival and the 3-year survival rate. The findings imply, at the castrated level, a significant difference in short-term survival between subgroups (see **Figure 3**).

## 4 Discussion

The safety and survival benefits of ADT in mHSPC have been established through numerous randomized controlled trials and clinical studies internationally. Testosterone management constitutes a critical component of holistic care strategies for metastatic prostate cancer patients. The foundational work of Huggins and Hodges established PCa as an androgen-dependent malignancy, demonstrating that reducing androgen levels or blocking androgen receptor signaling could inhibit PCa growth. Their research outlined two therapeutic approaches: (I) creating a hypoandrogenic environment through androgen deprivation and (II) administering supraphysiological androgen doses to induce hyperandrogenic conditions. Since then, PCa hormone therapy has undergone a long and tortuous progression from surgical and pharmacological castration for ADT to androgen antagonists and androgen receptor inhibitors (11). However, recent evidence has challenged the conventional understanding of testosterone–prostate cancer relationships. While some studies suggest reduced prostate cancer risk in men with low serum free testosterone (12), others indicate that neither elevated endogenous androgen levels nor testosterone replacement therapy in hypogonadal men significantly increases prostate cancer risk or disease aggressiveness (13). This study re-examines testosterone dynamics during ADT in mHSPC patients, specifically investigating the prognostic value of early (1 month after ADT) testosterone measurements.

Testosterone, the primary male sex hormone, is predominantly synthesized (approximately 90%) by Leydig cells located in the testicular interstitium between seminiferous tubules, with the adrenal glands contributing a minor portion (5%–10%) of total production. Notably, emerging evidence indicates that prostate tumor cells themselves may possess a limited testosterone-secreting capacity. Testosterone exists in two primary states: (I) bound form, comprising over 95% of total testosterone (TT) through association with sex hormone binding globulin (SHBG) and albumin, and (II) free testosterone (FT), representing 0.5%–3% of total circulating testosterone but constituting the biologically active fraction that directly interacts with target tissues. The combined pool of FT and albumin-bound testosterone, readily dissociable to exert physiological effects, is termed bioavailable testosterone (BT). While clinical practice typically relies on serum TT measurements, it is important to recognize that testosterone exerts its effects either through direct binding to androgen receptors in prostate tissue or via conversion to dihydrotestosterone by 5α-reductase, subsequently regulating prostate cancer development and progression (14, 15). Importantly, elevated SHBG levels demonstrate a causal association with reduced PCa risk, primarily through negative correlation with BT concentrations (16).

The demographic and baseline characteristics of the study population stratified by testosterone response status revealed no significant intergroup differences in Gleason score, T stage, prostate volume, initial total PSA, PSA density, perineural invasion, visceral metastasis, or hazard level (all *P* > 0.05). However, the testosterone non-response group demonstrated a significantly higher proportion of high tumor load compared to the response group (76.77% vs. 60.10%, *P* = 0.004). The testosterone response group exhibited superior ADT and testosterone management metrics, including longer cumulative treatment duration, reduced testosterone flash, fewer testosterone escape events, and greater treatment continuity. For ADT continuity, ADT dosing specifications, and T escapes, the time setting for testosterone response is 1 month, which seems to indicate a time lag. However, this phenomenon also reflects the relative stability and effectiveness of the current ADT treatment. Excellent depth reduction of testosterone in the early stages of ADT treatment often increases the continuity and stability of subsequent treatment. These findings may provide corroborating evidence that continuous androgen deprivation therapy (CADT) with long-acting formulations (3 months) represents a positive predictive factor for sustained testosterone early stage response. Current evidence indicates comparable efficacy between CADT and intermittent ADT (IADT) in mHSPC regarding overall survival and quality of life (17, 18). However, IADT poses significant challenges to testosterone control, with nearly half of the patients exceeding 20 ng/dL within 1 month of treatment cessation, often reaching mean levels twice the castration threshold. These elevations frequently remain undetected without systematic monitoring (19). Real-world data confirm that long-acting LHRH analogs (goserelin, triptorelin, leuprolide) effectively maintain castrate testosterone levels with favorable tolerability profiles in advanced PCa (20, 21).

An analysis of progression to mCRPC revealed significantly shorter time intervals in the testosterone non-response group compared to the response group (13.38 ± 8.88 vs. 20.40 ± 11.91 months, *P* < 0.001). However, among testosterone response patients, no significant difference emerged between low (20.59 ± 11.91 months) and ultra-low (20.86 ± 12.19 months, *P* = 0.876) response subgroups. These findings demonstrate that while achieving castrate testosterone levels significantly influences disease progression in mHSPC, the depth of testosterone suppression beyond castration thresholds does not substantially affect cancer progression. Our results corroborate emerging evidence that lower castration thresholds (T < 32 ng/dL) provide superior predictive value for mCRPC progression compared to conventional standards (T < 50 ng/dL) (22) and further support consideration of even more stringent thresholds (T < 20 ng/dL) for optimal PCa monitoring. The subgroup analysis revealed no statistically significant differences in demographic and baseline characteristics except for testosterone flash (*P* = 0.007), a phenomenon directly attributable to insufficient depth of testosterone suppression. Other clinical variables including tumor load, ADT continuity, and dosing specifications showed no intergroup differences (all *P* > 0.05), reinforcing that once castrate levels are achieved, additional testosterone reduction does not significantly affect the progression timelines to mCRPC. The survival analysis demonstrated comparable long-term outcomes between response groups, with no significant differences in overall survival (*P* = 0.114) or 5-year survival rates (*P* = 0.320). However, the testosterone response group showed superior 3-year survival (*P* = 0.024), suggesting short-term survival benefits. Further stratification within responsive patients revealed significant survival advantages for ultra-low versus low responders in both overall survival (*P* = 0.010) and 3-year survival (*P* = 0.001), though the 5-year survival rates remained comparable (*P* = 0.107). These findings reflect real-world clinical patterns where advanced PCa management increasingly utilizes multimodal approaches including novel endocrine therapies, chemotherapy, radiotherapy, brachytherapy, targeted agents, and immunotherapy, often in triple or quadruple combinations that can achieve superior progression-free survival (23, 24). Nevertheless, optimal treatment sequencing strategies to maximize cumulative survival benefits and prevent early resistance remain undefined (25). The observed short-term survival advantage (3 years) for ultra-low testosterone responders, particularly significant given our 1-month assessment timeframe, suggests that profound early testosterone suppression may confer clinical benefits despite the dynamic, fluctuating nature of testosterone levels over extended periods. Importantly, patients with high tumor load mPCa achieving serum testosterone <20 ng/dL within one month of ADT initiation demonstrated improved progression-free and overall survival. These findings align with evidence that delayed achievement of deep testosterone suppression correlates with poorer prognosis (26). A large-scale Japanese cohort data showed median progression-free survival of 44.5 versus 16.1 months and median overall survival of 103.2 versus 62.7 months for low versus high tumor load patients, respectively (27).

Testosterone-responsive patients undergoing CADT and IADT demonstrate significant differences in testosterone levels (28), potentially attributable to differential treatment responses influenced by age, racial background, and ADT duration (29–31). During IADT intervals, most advanced PCa patients fail to recover baseline or gonadal-normal testosterone levels, with approximately 10% maintaining castrate testosterone concentrations up to two years after treatment cessation (32, 33). These findings underscore the necessity for standardized testosterone monitoring protocols during the metastatic hormone-sensitive phase. Initial assessment should precede radiotherapy or endocrine interventions to establish baseline values, as pre-treatment testosterone levels demonstrate prognostic significance for disease progression. Critical evaluation at 1 month after ADT initiation determines whether castration thresholds are achieved: testosterone levels >50 ng/dL indicate treatment failure requiring regimen modifications (e.g., switching LHRH analogs, though surgical castration is generally not recommended). Subsequent monitoring should verify sustained castration while assessing treatment adherence. Collectively, these observations position testosterone as an accessible, cost-effective biomarker with independent prognostic value in mHSPC. Its integration with PSA monitoring may provide synergistic guidance for clinical decision-making regarding treatment selection, modification, and intensification (34–37).

The mechanistic relationship between testosterone and prostate cancer progression remains controversial, particularly regarding whether androgen stimulation follows a threshold effect or a dose–response pattern. Previous studies have indicated that the ability of testosterone to stimulate the growth of prostate cancer cells is limited and reaches a maximum at low concentrations of testosterone, referred to as the saturation model, with a saturation point of T_max_ = 250 ng/dL (38, 39). This nonlinear relationship has inspired innovative therapeutic approaches utilizing supraphysiological testosterone levels to delay disease progression in advanced prostate cancer, a strategy termed biphasic androgen therapy (BAT). Originally developed for patients with CRPC, BAT involves rapid cycling between supraphysiological and near-castrate testosterone levels. This approach demonstrates multiple clinical benefits: (a) established safety in asymptomatic mCRPC patients, (b) delayed cancer progression, (c) sustained prostate-specific antigen suppression, and (d) restored sensitivity to subsequent combined androgen blockade therapy (40, 41).

Although the pathophysiology of benign prostatic hyperplasia and malignant prostate cancer is well characterized, fundamental understanding of how normal and neoplastic prostate tissues (predominantly adenocarcinoma) respond physiologically to serum testosterone remains limited. This knowledge gap is particularly evident regarding the tumor percentage within heterogeneous prostate specimens and the testosterone responsiveness of residual cancerous or hyperplastic tissues following transurethral resection or radical prostatectomy. Notably, the anatomical proximity between testosterone-producing testicular tissue and PSA-secreting prostate tissue suggests potential mechanistic interplay or “neighborhood effects” warranting further investigation. Testosterone management represents a critical component throughout the prostate cancer care continuum, from initial diagnosis through treatment assessment and prognostic evaluation. Failure to achieve or maintain castrate testosterone levels (testosterone escape) may result from multiple factors including dietary influences, metabolic status, non-adherence, or administration irregularities. Serial testosterone monitoring should be implemented at key clinical junctures (diagnosis, recurrence, metastasis) and therapeutic decision points (treatment combination/modification, castration resistance) to inform management strategies. The epidemiological characteristics of prostate cancer exhibit regional and ethnic variations. Similarly, the response to testosterone in ADT may also exhibit regional and ethnic variations, and these variations may lead to differences in clinical treatment responses. Therefore, it is necessary to further discuss and improve the current standard clinical diagnostic and treatment model in conjunction with real-world conditions. The increasing trends in prostate cancer incidence and mortality rates in Asia are multifactorial, with possible explanatory factors including diet, lifestyle, and statistical methods (42). In addition to regional factors, prostate cancer specificity also involves racial susceptibility, particularly among African men, who have higher incidence rates and poorer outcomes (43). In the complex real-world setting, potential interactions between confounding factors and hormones, including androgen-thyroid hormone and adipose tissue–prostate cancer interactions, contribute to the complex hormonal microenvironment of prostate cancer, thereby influencing the response to subsequent endocrine therapy (44, 45). Five fundamental questions require elucidation regarding testosterone’s role in prostate cancer, namely: (I) the correlation between endogenous testosterone levels and prostate cancer risk, tumor load, and Gleason score, (II) the relationship between endogenous testosterone and disease progression dynamics, (III) the clinical relevance of bioavailable testosterone versus total testosterone, particularly in advanced disease states characterized by negative nitrogen balance, (IV) The optimal testosterone threshold for advanced prostate cancer management—whether lower non-castrate or higher supra-physiological levels provide superior outcomes, and (V) the long-term prognostic value of BAT in advanced prostate cancer.

Recent therapeutic advances, including combination regimens (triplet or quadruplet therapy), have demonstrated significant clinical benefits over traditional approaches in high tumor load mHSPC. The integration of novel endocrine therapies (abiraterone, apalutamide, enzalutamide) and cytotoxic agents (docetaxel, cisplatin) with ADT introduces new complexities in testosterone management. Future investigations should systematically evaluate how ADT-based combination therapies, incorporating diverse endocrine agents, chemotherapy, and radiotherapy, influence testosterone dynamics during standardized monitoring and how testosterone levels affect advanced prostate cancer incidence, progression, and prognosis. Notably, the potential reproductive toxicity of chemotherapeutic agents and radiotherapy on testicular and adrenal function may synergize with ADT by further suppressing endogenous testosterone production. In contemporary clinical practice, standardized ADT regimens typically achieve near-undetectable testosterone levels (T < 0.01 ng/dL) early in treatment, with minimal escape or fluctuation. Under these conditions, the clinical relevance of traditional castration thresholds (20 ng/dL or 50 ng/dL) becomes uncertain. However, long-term monitoring remains crucial, as late testosterone escape may herald emerging resistance to anticancer therapies. To fully characterize these phenomena, multi-institutional collaborations are needed to establish comprehensive testosterone profiling across mHSPC and mCRPC.

This study reaffirms that ADT constitutes the cornerstone treatment for prostate cancer. Our findings indicate that after testosterone reaches the standard castrated level (T < 50 ng/dL) after 1 month of ADT, the reduction of testosterone to ultra-low level (T < 20 ng/dL) can be used as a reference for better prognosis and adjustment of treatment for prostate cancer patients. While the precise relationship between testosterone and prostate cancer remains incompletely characterized, our evidence strongly establishes serum testosterone dynamics as a crucial determinant of prostate cancer progression risk.

*The future of hormone therapy for prostate cancer is undoubtedly full of challenges and opportunities, contradictions, and denials. But firmly believe the road ahead is long but full of promise.*

### 4.1 Innovations in this study

This study systematically examines testosterone’s role in mHSPC progression, revealing temporal associations between androgen dynamics and disease evolution. By redefining the relationship between endocrine (testosterone) and genitourinary malignancy (prostate cancer), this study enables improved risk stratification and provides a foundation for early intervention and precision medicine approaches in mHSPC.

### 4.2 Limitations in this study

1. As a dual-center study conducted in eastern and western China, our investigation utilized a relatively limited sample size (*N* = 366). This constraint stems from two factors: stringent inclusion/exclusion criteria necessitating testosterone monitoring and declining proportions of metastatic prostate cancer cases due to widespread PSA screening implementation.

2. This study reliance on total testosterone measurements, rather than free or bioavailable testosterone, introduces potential analytical limitations. However, this approach reflects real-world clinical practice where total testosterone remains the standard metric due to technical accessibility and cost-effectiveness. Importantly, total testosterone demonstrates a strong correlation with free and bioavailable fractions, justifying its use for assessing disease progression, prognostic evaluation, and clinical decision-making in mHSPC.

3. Inter-institutional variability in testosterone assays and incomplete quality control standardization may introduce measurement bias. To mitigate this, we implemented standardized monitoring protocols, ensuring near-optimal consistency in testing conditions, methodologies, and timing across participating centers.

While dual-center designs inherently limit generalizability compared to multi-center trials, our study provides foundational evidence for future collaborative efforts. This study proposes expanding this research through a cross-regional, multi-center consortium across Anhui and Xinjiang provinces to conduct large-scale, long-term studies establishing standardized treatment paradigms for advanced prostate cancer. Such initiatives would advance the standardization, normalization, and effective management of endocrine therapy throughout the mHSPC disease continuum.

## Data Availability

The original contributions presented in the study are included in the article/supplementary material. Further inquiries can be directed to the corresponding author.
